# Georeferencing of sepsis in São Paulo

**DOI:** 10.1186/cc12666

**Published:** 2013-06-19

**Authors:** F Colombari, D Diament, AS Cypriano, LF Lisboa, BF Cardoso dos Santos, MC Neto, E Silva

**Affiliations:** 1 Hospital Israelita Albert Einstein, Morumbi, São Paulo, SP, Brazil

## Introduction

Morbidity and lethality in sepsis varies according to demographic and socioeconomic factors, which can be linked to geographic distribution of populations. The objective of this study is to relate sepsis deaths with geographic distribution in the city of São Paulo, Brazil.

## Methods

Death certificates for sepsis and sepsis-related infections (pneumonia, urinary tract infections, meningitis, skin and soft tissue infections, peritonitis, multiple organ failure) from 2004 to 2009 in the city of São Paulo, Brazil, were searched. Patients were distributed according to sex, age, main cause of death, secondary cause of death, residence address, and death location. The Human Development Index (HDI) was used to compare city districts. Health institutions were identified as public or private administered according to Ministry of Health registration.

## Results

The number of deaths increased with age, but there is no difference between sexes for the whole population studied. However, in younger age groups (up to 18 years and from 18 to 64 years) deaths were more frequent for males than females (53.9 × 46.1% and 61 × 39%, respectively). In the age group older than 65 years there were 46.3% of deaths in males and 53.7% for females. Deaths occurred homogeneously in all city districts, but its frequency was larger in districts with higher HDI. Death location was 93.73% in hospitals and 5.47% in dwellings. From the identifiable institutions where deaths occurred, 52.4% were public, 46.4% were private and 1.2% were nonidentifiable. The mean death charge was 79% higher in public institutions compared with private ones. There was no visible difference in death distribution according to age and residence address. Comparing patients' residence address with death location showed a concentration near the closest hospital, without difference between private or public hospitals. There was a higher proportion of deaths for the age group older than 65 years in private hospitals. For the age group between 19 and 64 years, the proportion of deaths was slightly higher in public hospitals. The main cause of death was pneumonia, followed by sepsis, multiple organ failure, intra-abdominal infections, meningitis, skin and soft tissue infections and urinary tract infections. Distributing main death causes by age groups, patients older than 65 years died more over pneumonia, sepsis and multiple organ failure. Patients up to 18 years died more over urinary tract infections and meningitis. The age group between 19 and 64 years was distributed fairly amongst all death causes.

## Conclusion

Georeferencing is a potent tool for epidemiological studies. Deaths occurred homogeneously in all city districts, but the frequency was larger in districts with higher HDI. As expected, older patients died in greater numbers, affected by respiratory tract infections, sepsis and multiple organ failure. Deaths occurred mainly in the public health system.

